# Cyr61 Expression is associated with prognosis in patients with colorectal cancer

**DOI:** 10.1186/1471-2407-14-164

**Published:** 2014-03-07

**Authors:** Dongjun Jeong, Suhak Heo, Tae Sung Ahn, Sookyoung Lee, Soyoung Park, Hyungjoo Kim, Doosan Park, Sang Byung Bae, Sung Soo Lee, Moon Soo Lee, Chang-Jin Kim, Moo Jun Baek

**Affiliations:** 1 Department of Pathology, College of Medicine, Soonchunhyang University, 31 soonchunhyang 6 gil, Dongnam-gu, Cheonan, Chungcheongnam-do 330-722, Republic of Korea; 2 Department of Biochemistry, College of Medicine, Soonchunhyang University, 31 soonchunhyang 6 gil, Dongnam-gu, Cheonan, Chungcheongnam-do 330-722, Republic of Korea; 3 Department of Surgery, College of Medicine, Soonchunhyang University, 31 soonchunhyang 6 gil, Dongnam-gu, Cheonan, Chungcheongnam-do 330-722, Republic of Korea; 4 Department of Oncology, College of Medicine, Soonchunhyang University, 31 soonchunhyang 6 gil, Dongnam-gu, Cheonan, Chungcheongnam-do 330-722, Republic of Korea; 5 Department of Preventive Medicine, College of Medicine, Soonchunhyang University, 31 soonchunhyang 6 gil, Dongnam-gu, Cheonan, Chungcheongnam-do 330-722, Republic of Korea; 6 Soonchunhyang Medical Science Research Institute, College of Medicine, Soonchunhyang University, 31 soonchunhyang 6 gil, Dongnam-gu, Cheonan, Chungcheongnam-do 330-722, Republic of Korea

**Keywords:** Colorectal cancer, Cyr61, Immunohistochemistry, Prognosis

## Abstract

**Background:**

Cysteine-rich 61 (Cyr61), a member of the CCN protein family, possesses diverse functionality in cellular processes such as adhesion, migration, proliferation, and survival. Cyr61 can also function as an oncogene or a tumour suppressor, depending on the origin of the cancer. Only a few studies have reported Cyr61 expression in colorectal cancer. In this study, we assessed the Cyr61 expression in 251 colorectal cancers with clinical follow up.

**Methods:**

We examined Cyr61 expression in 6 colorectal cancer cell lines (HT29, Colo205, Lovo, HCT116, SW480, SW620) and 20 sets of paired normal and colorectal cancer tissues by western blot. To validate the association of Cyr61 expression with clinicopathological parameters, we assessed Cyr61 expression using tissue microarray analysis of primary colorectal cancer by immunohistochemical analysis.

**Results:**

We verified that all of the cancer cell lines expressed Cyr61; 2 cell lines (HT29 and Colo205) demonstrated Cyr61 expression to a slight extent, while 4 cell lines (Lovo, HCT116, SW480, SW620) demonstrated greater Cyr61 expression than HT29 and Colo205 cell lines. Among the 20 cases of paired normal and tumour tissues, greater Cyr61 expression was observed in 16 (80%) tumour tissues than in normal tissues. Furthermore, 157 out of 251 cases (62.5%) of colorectal cancer examined in this study displayed strong Cyr61 expression. Cyr61 expression was found to be associated with pN (p = 0.018). Moreover, Cyr61 expression was associated with statistically significant cancer-specific mortality (p = 0.029). The duration of survival was significantly lesser in patients with Cyr61 high expression than in patients with Cyr61 low expression (p = 0.001). These results suggest that Cyr61 expression plays several important roles in carcinogenesis and may also be a good prognostic marker for colorectal cancer.

**Conclusions:**

Our data confirmed that Cyr61 was expressed in colorectal cancers and the expression was correlated with worse prognosis of colorectal cancers.

## Background

Cysteine-rich 61 (Cyr61) is a member of the CCN (**C**yr61/**C**TGF/**N**ov) protein family, which consists of Cyr61 (CCN1); connective tissue growth factor (CTGF/CCN2), nephroblastoma-overexpressed (Nov/CCN3); and Wnt-induced secreted proteins 1, 2 and 3 (Wisp-1/CCN4, Wisp-2/CCN5, and Wisp-3/CCN6, respectively). These CCN proteins are involved in multiple functional pathways, including mitogenesis, cellular adhesion, migration, cell survival, differentiation, angiogenesis, and wound healing [1]. Among the various CCN proteins, a majority of studies have been conducted on Cyr61 or CCN1 with reference to cancer. Cyr61 is expressed by all types of vascular cells, participating in such diverse cellular processes as adhesion, migration, proliferation, and survival [2,3]. Notably, an important role for Cyr61 in the processes of angiogenesis and vascularization is being seen. Neovascularization of tumours is one of the most important functions of tumour growth factors; thus, Cyr61 participates in tumour progression by neovascularization. In addition to angiogenesis, CCN1 can promote cancer cell proliferation, invasion, survival, and metastasis [4,5]. Overexpression of Cyr61 is associated with the growth and progression of breast cancer [6], ovarian cancer [7], gastric cancer [8,9], and glioma [10,11]. In contrast, Cyr61 has also been shown to function as a tumour suppressor in prostate cancer [12], uterine leiomyoma [13], non-small cell lung cancer [14,15], and endometrial cancer [16]. Cyr61 may exert different, even opposing, functions in tumorigenesis, depending on the cell types.

Colorectal cancer (CRC) is one of the commonest cancers worldwide and was ranked second in 2010 for cancer incidence in South Korea [17]. At least 3 primary molecular pathways involved in the progression of CRC have been identified, including the prevalent chromosomal instability (CIN) pathway (identified in up to 80% of cases), the CpG island methylator phenotype (CIMP) pathway, and microsatellite instability (MSI), resulting from the DNA mismatch repair (MMR) gene [18]. However, the precise mechanism underlying colorectal carcinogenesis remains unclear.

More recently, studies have focused primarily on molecular biomarkers to indicate progression of a disease or susceptibility of the disease to a given treatment [19].

Cyr61 plays important roles in carcinogenesis, either as an oncogene or as a tumour suppressor gene, depending on the cancer type. The purpose of our study is to clarify whether Cyr61 functions as an oncogene or as an anti-oncogene in CRC and to determine whether there is a correlation between Cyr61 expression and clinicopathological parameters, including prognosis. We also verified that Cyr61 is a relevant biomarker of CRC progression.

## Methods

### Patients and samples

We obtained formalin-fixed, paraffin-embedded specimens from 251 patients with primary CRC. All patients with cancer underwent surgery from 2002 to 2007. Clinicopathological data including age, sex, lymph node metastasis, American Joint Committee on Cancer (AJCC) TNM classification, and tumour differentiation were recorded (Table 1). Our work was approved by the Institutional Review Board of the College of Medicine, Soonchunhyang University.

**Table 1:** Clinicopathological factors of the study

**Clincopathological factors**	**Number**
**Sex**	
Male	147
Female	104
**pT stage**	
1	10
2	41
3	171
4	29
**pN stage**	
0	136
1	75
2	40
**Vascular invasion**	
0	219
1	32
**Lymphatic invasion**	
0	212
1	39
**TNM stage**	
I	35
II	98
III	105
IV	13

### Western blot assay

Cancer cells were washed with PBS and lysed using the Pro-Prep protein extraction kit (Intron, Seoul, South Korea) at 4°C overnight. The supernatant containing protein was collected after centrifugation. Protein concentrations were determined using a BCA Protein Assay Kit (Thermo Scientific, Waltham, MA, USA). Cell lysate samples (30 μg) were loaded onto 10% SDS-PAGE, separated at 110 V for 2 h, and transferred to a PVDF membrane with the Trans-Blot Turbo system (Bio-Rad Laboratories, Hercules, CA, USA). The membrane was blocked for 1 h in blocking buffer consisting of 5% non-fat skim milk/1X TBS-T (Tris-buffered saline with 0.1% Tween 20) for 1 h at room temperature, followed by incubation with primary monoclonal anti-Cyr61 antibody at 4°C overnight, diluted in 1X TBS-T (1:1000; R&D Systems, Minneapolis, MN, USA). After a 5 times wash with TBS-T, the membrane was incubated with a goat anti-mouse secondary antibody in 1X TBS-T (1:80,000 dilution; Sigma Chemicals, St. Louis, MO, USA) for 1 h at room temperature. The membranes were washed 4–5 times in 1X TBS-T for 1 h. The signal was detected using an enhanced chemiluminescence kit (ECM solution; Advansta, Menlo Park, CA, USA) and Molecular Imager ChemiDoc XRS + System (Bio-Rad Laboratories). Beta-actin was used as the loading control. The relative Cyr61 expression was measured by Image Densitometer (Bio-Rad, Laboratories) with beta-actin normalization. The relative Cyr61 expressions in tumour tissues compared to those in normal tissues were calculated by tumour/normal ratio.

### Tissue microarray

Tissue microarrays (TMAs) were assembled from 10% neutral buffered formalin-fixed paraffin-embedded tissues by using a 2-mm-diameter punch (UNITMA, Unitech Science, Seoul, South Korea). Tissue microarrays were constructed by obtaining primarily duplicate cores of formalin-fixed paraffin-embedded tumour specimens and re-embedding the cores in an arrayed recipient paraffin block. A TMA block contains 60 cores from 30 samples. Samples obtained from the same tumour were staggered in the array, and a map was created for later identification of the identity of the individual cores.

### Immunohistochemical analysis

Four-micrometer-thick sections were sliced onto Silane Coated Micro Slides (Muto Pure Chemicals Corp., Tokyo, Japan) and incubated at 60°C for 2 h. The slides were then deparaffinized by application of xylene and incubation (5 min ×3) at room temperature. Sections were hydrated by application of graded alcohol, and endogenous peroxidase activity was quenched by incubating the sections in 0.3% H_2_O_2_ in methanol for 30 min at room temperature. After washing the slides in phosphate-buffered saline (PBS; 5 min × 2), antigen retrieval was performed by heating the slides in citrate buffer (0.01M, pH 6.0) using a microwave in a pressure cooker for 15 min. After heating, the samples were allowed to cool off for 2 h at room temperature followed by washing with PBS (3 min × 2). Immunohistochemical (IHC) analysis was performed using Cyr61 primary antibody (Abcam, Cambridge, UK) with incubation for 2 h at room temperature with an UltraVision Quanto Detection System HRP DAB (Lab Vision Corp., Fremont, CA, USA) according to the manufacturer’s instructions.

### IHC data analysis

The Cyr61-stained tissue cores were examined by 2 independent observers (CJK and DJJ), and a consensus score was determined for each specimen. A positive reaction was scored into 4 grades, according to the intensity of the staining: 0, 1+, 2+, and 3+. The percentages of Cyr61-positive cells were also scored into 4 categories: 0 (0%), 1 (1–33%), 2 (34–66%), and 3 (67–100%). The final score, calculated as the product of the intensity score multiplied by the percentage score, was classified as follows: 0 for negative; 1–3 for weak; 4–6 for moderate; and 7–9 for strong. Samples with a final score ≤3 were grouped together as Cyr61 expression negative while those with a score ≥4 were grouped together as Cyr61 expression positive.

### Statistical analysis

The data were analysed using commercially available software (SPSS 18.0; SPSS; Chicago, IL, USA). The clinical and pathologic factors that were evaluated included pT (1, 2, 3, and 4), pN (0, 1, and 2), vascular invasion, lymphatic invasion, and TNM stage classification (I, II, III, and IV). For investigating the relationships between Cyr61 expression and the clinicopathological factors, chi-square tests were performed. A *p* value, two-sided test was also performed; values less than 0.05 were considered statistically significant for all analyses. The clinical and pathologic variables and Cyr61 expression were considered for survival analysis using the Kaplan-Meier method. The statistical significance was set at a p value less than 0.05 and assessed by the log-rank test. Hazard ratio (HR) and 95% confidence interval (95% CI) were estimated from Cox proportional hazard models. For final multivariable Cox regression models, all covariates were controlled.

## Results

### Cyr61 protein expression in CRC cell lines and normal-cancer paired CRC tissues by western blot

**Figure 1:**
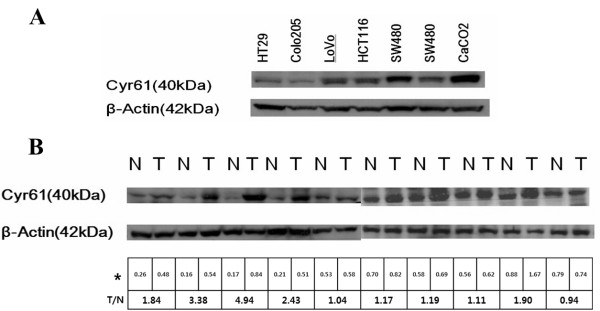
**Western blot analysis of Cyr61 expression. (A)** All of the cancer cell lines expressed Cyr61. **(B)** Representative 10 pairs of normal and tumour tissue. Greater Cyr61 expressions were observed in tumour tissues than in normal tissues. The relative Cyr61 expression was measured by densitometry with beta-actin normalization (*). The relative Cyr61 expressions in tumour tissues compared to those in normal tissues were calculated by tumour/normal ratio (T/N).

We examined Cyr61 expression in 6 CRC cell lines by Western blot. All 6 cancer cell lines expressed Cyr61; 2 cell lines (HT29 and Colo205) demonstrated slight Cyr61 expression, while 4 cell lines (Lovo, HCT116, SW480, SW620) demonstrated greater Cyr61 expression than the HT29 and Colo205 cell lines (Figure 1A). Furthermore, we examined Cyr61 expression in 20 sets of paired normal and CRC tissues by western blot. Among the 20 normal and tumour tissue pairs, greater Cyr61 expression was observed in tumour tissues versus normal tissues in 16 cases (80%) (Figure 1B).

### Cyr61 expression in CRC tissue by Immunohistochemistry

**Figure 2:**
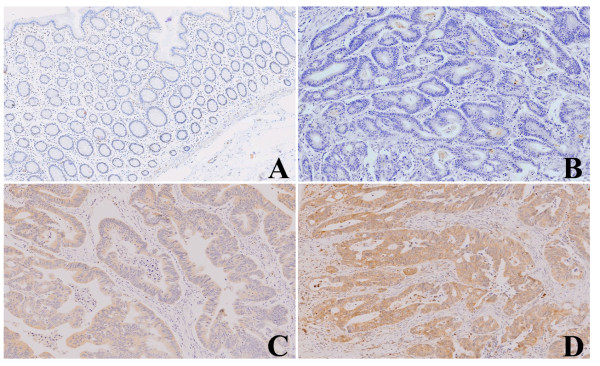
**Cyr61 expressions in tissues by immunohistochemistry. (A)** Normal glands in the colorectum demonstrate negative Cyr61 expression. **(B)** Some colorectal cancer shows negative Cyr61 expression. **(C)** Cyr61 is expressed in mild degree in the carcinoma. **(D)** Cyr61 is expressed in severe degree in the carcinoma.

**Figure 3:**
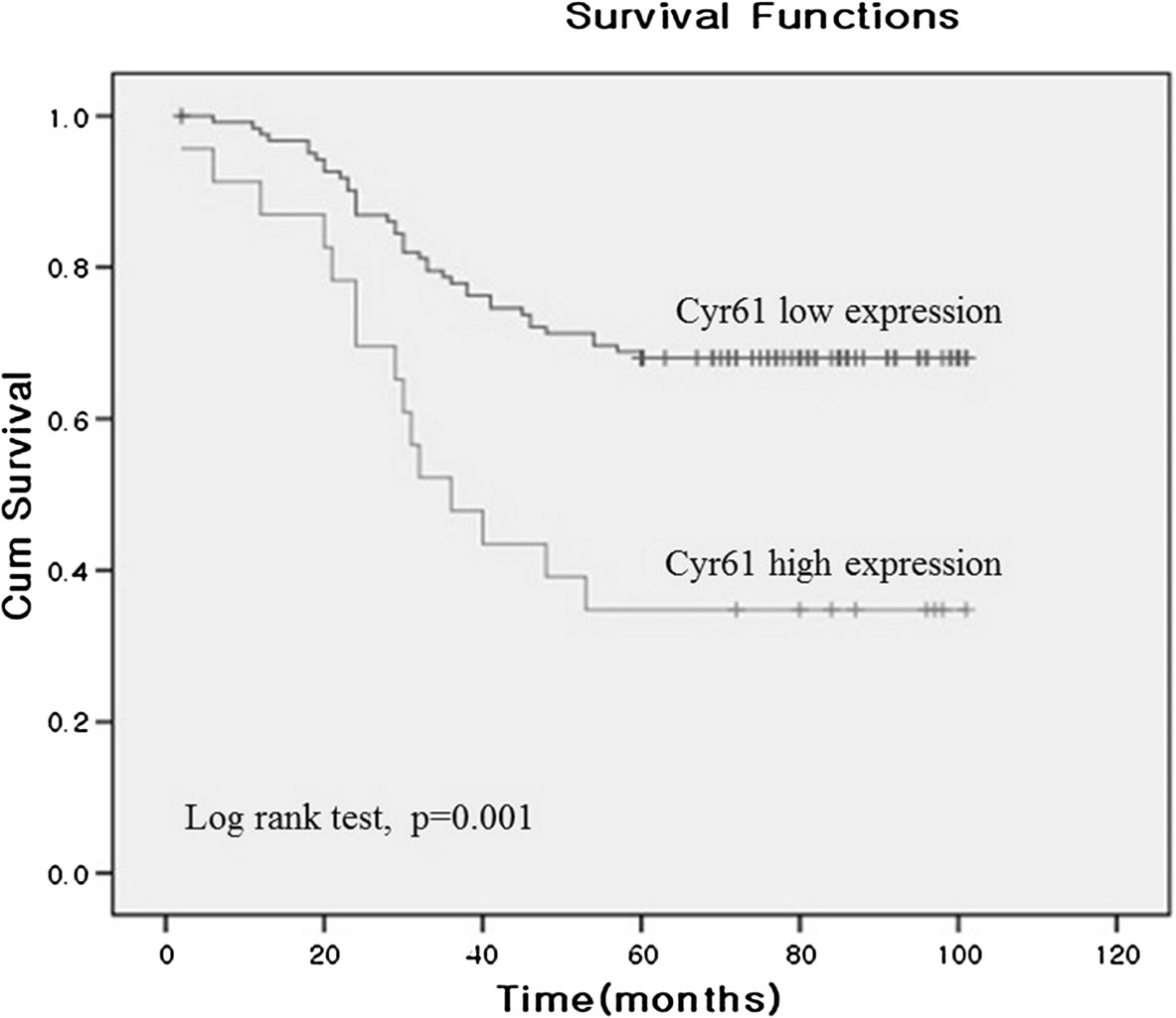
**Survival curves from the time of diagnosis for colorectal cancer patients by Cyr61 expression.** The duration of survival was significantly lesser in patients with Cyr61 high expression than in those with Cyr61 low expression.

Cyr61 was not expressed in most normal tissues. In tumour tissue, Cyr61 was expressed with a wide range of intensity, ranging from negative to severe staining (Figure 2A-D). Among the 251 cases, a positive result for IHC Cyr61 staining was observed in 157 samples (62.2%) (Table 2). The frequency of Cyr61 expression was 5.1%, 12.7%, 66.9%, and 15.3% for pT stages 1, 2, 3, and 4, respectively. Positive Cyr61 expression was significantly related with the pT stage (p = 0.021). The frequencies of Cyr61 expression for pN stages 0, 1, and 2 were 46.5%, 35.7% and 17.8%, respectively; these results were statistically significant (p = 0.006). Furthermore, the frequencies of Cyr61 expression were 13.4% and 16.6% when examining vascular invasion and lymphatic invasion, respectively; however, these results were not statistically significant (p = 0.700 and p = 0.563, respectively). However, the frequencies of Cyt61 expression for TNM stages I, II, III, and IV were 10.8%, 32.5%, 50.3%, and 6.4% respectively (p = 0.001); the results were statistically significant (Table 2). Age, sex, pT stage, venous invasion, lymphatic invasion, tumour differentiation were not related with prognosis by univariate and multivariate Cox hazard regression analysis. pN stage 1 was found to be associated with prognosis in multivariate analysis (HR, 2.02; 95% CI, 1.04–3.94; p = 0.038). Additionally, pN stage 2 was associated with prognosis both in univariate analysis (HR, 2.90; 95% CI, 1.44–5.83; p = 0.003) and in multivariate analysis (HR, 2.53; 95% CI, 1.20–5.32; p = 0.015) (Table 3). Moreover, strong Cyr61 expression was found to be prognostic factor both in univariate analysis (HR, 2.56; 95% CI, 1.29–5.08; p = 0.007) and in multivariate analysis (HR, 2.31; 95% CI, 1.09–4.89; p = 0.029), as determined by Cox hazard regression analysis (Table 3). The duration of survival was significantly lesser in patients with Cyr61 high expression than in those Cyr61 low expression (p = 0.001) (Figure 3).

**Table 2:** Comparison of clinicopathological factors and Cyr61 expression

**Clincopathological factors**	**Cyr61**		**Total (%)**	**p**
	**Positive N = 157 (%)**	**Negative N = 94 (%)**		
**Age, years, mean (SD)**	62.2 (12.2)	65.2 (11.5)	63.3 (12.0)	0.061
**Sex**				0.296
**Male**	88 (56.1)	59 (62.8)	147 (58.6)	
**Female**	69 (43.9)	35 (37.2)	104 (41.4)	
**pT stage**				0.021
**1**	8 (5.1)	2 (2.1)	10 (4.0)	
**2**	20 (12.7)	21 (22.3)	41 (16.3)	
**3**	105 (66.9)	66 (70.2)	171 (68.1)	
**4**	24 (15.3)	5 (5.3)	29 (11.6)	
**pN stage**				0.006
**0**	73 (46.5)	63 (67.0)	136 (54.2)	
**1**	56 (35.7)	19 (20.2)	75 (29.9)	
**2**	28 (17.8)	12 (12.8)	40 (15.9)	
**Vascular invasion**				0.700
**0**	136 (86.6)	83 (88.3)	219 (87.3)	
**1**	21 (13.4)	11 (11.7)	32 (12.7)	
**Lymphatic invasion**				0.563
**0**	131 (83.4)	81 (86.2)	212 (84.5)	
**1**	26 (16.6)	13 (13.8)	39 (15.5)	
**TNM stage**				0.001
**I**	17 (10.8)	18 (19.1)	35 (13.9)	
**II**	51 (32.5)	47 (50.0)	98 (39.0)	
**III**	79 (50.3)	26 (27.7)	105 (41.8)	
**IV**	10 (6.4)	3 (3.2)	13 (5.2)	

**Table 3:** Cox hazards regression analysis of the clinicopathological factors

**Clinicopathological factors**	**Univariate analysis**		**Multivariate analysis**	
	**Hazard ratio (95% CI)**	**P**	**Hazard ratio (95% CI)**	**P**
**Age**	1.01 (0.99–1.03)	0.514	1.00 (0.98–1.03)	0.858
**Sex (0 = male, 1 = female)**	1.03 (0.60–1.77)	0.917	1.20 (0.66–2.20)	0.548
**pT stage**				
**1**	1.00		1.00	
**2**	1.09 (0.24–5.03)	0.916	0.93 (0.18–4.86)	0.927
**3**	1.13 (0.27–4.70)	0.864	0.99 (0.19–4.88)	0.967
**4**	3.44 (0.68–17.78)	0.140	2.15 (0.34–13.86)	0.419
**pN stage**				
**0**	1.00		1.00	
**1**	1.83 (0.99–3.40)	0.056	2.02 (1.04–3.94)	0.038
**2**	2.90 (1.44–5.83)	0.003	2.53 (1.20–5.32)	0.015
**Venous invasion**	1.00 (0.45–2.21)	0.991	0.67 (0.23–1.96)	0.463
**Lymphatic invasion**	1.84 (0.98–3.44)	0.057	1.97 (0.79–4.90)	0.143
**Differentiation**				
**Well**	1.00		1.00	
**Moderate**	0.70 (0.36–1.40)	0.298	0.61 (0.28–1.30)	0.198
**Poor**	0.86 (0.24–3.08)	0.817	0.75 (0.18–3.09)	0.692
**Cyr61 Expression**				
**Negative**	1.00		1.00	
**Negative to mild expression**	0.89 (0.48–1.67)	0.721	0.86 (0.44–1.70)	0.662
**Moderate to severe expression**	2.56 (1.29–5.08)	0.007	2.31 (1.09–4.89)	0.029

CI, confidence interval.

## Discussion

Cyr61 is a multifunctional matricellular protein that performs various essential roles in cardiovascular development during embryogenesis, as well as regulates would healing, inflammation, and fibrogenesis [20]. Aberrant Cyr61 expression is associated with the development of various cancers and diseases linked to chronic inflammation. Cyr61 promotes diverse, and sometimes opposing, cellular responses. Structurally, CYR61 protein consists of an N-terminal secretory peptide followed by 4 conserved domains with sequence homologies to insulin-like growth factor-binding protein (IGFBP), von Willebrand factor C repeat (VWC), thrombospondin type I repeat (TSR), and a carboxyl-terminal (CT) domain that contains a cysteine knot motif [1]. A nonconserved central hinge region bisects the protein into 2 halves that bind to distinct receptors and induce diverse and disparate cellular responses. Early studies on Cyr61 protein showed that it is tightly associated with the extracellular matrix (ECM) [21] and supports cellular adhesion by direct binding to integrin receptors [22,23]. Cyr61 functions as a matricellular protein rather than a growth factor to induce cell proliferation [24]. Cyr61 also demonstrates various disparate cellular activities; for example, it promotes cellular survival, yet triggers cellular apoptosis; it enhances cell proliferation, yet induces cell-cycle arrest; and it promotes tumour growth, yet suppresses tumorigenesis in varying contexts, depending on the cell type. These disparate Cyr61 activities are attributed to its interaction with distinct integrins and heparan sulphate proteoglycans (HSPGs) on the surface of both endothelial cells and stromal fibroblasts. These Cyr61 activities stimulate both epithelial-mesenchymal transition and invasion of certain cancer cells [4,5,25]. Moreover, Cyr61 induces angiogenesis, which is essential for the supply of oxygen and nutrients to nourish tumour growth [26]. In addition to angiogenesis, Cyr61 promotes cancer cell proliferation, invasion, survival, and metastasis [4,5,27]. Significant correlation between Cyr61expression and tumour stage, tumour size, lymph node positivity, and poor prognosis have been reported in several types of cancers, including breast cancer [3,28-31], prostate cancer [32], glioma [33], gastric cancer [9], and oral squamous cell carcinoma [34]. However, only a few studies have been performed on the relationship between Cyr61 expression and CRC initiation and/or progression. A single report by Ladwa et al. [35] was retrieved in a worldwide search of journals published in English; they reported that Cyr61 was expressed in 82% (23/28) of CRCs examined and that Cyr61 expression was associated with more advanced clinical stages. In our study, all 7 CRC cell lines expressed Cyr61 with varying intensities. Five cell lines exhibited relatively strong expression levels of Cyr61, while 2 cells lines expressed lower levels of Cyr61. Furthermore, among the 20 sets of paired normal and cancer tissues, the tumour tissues exhibited stronger Cyr61 expression than paired normal tissues in 16 cases (80%). This result suggests that Cyr61 plays several roles in colorectal carcinogenesis, a hypothesis supported by the Cyr61 expression levels observed by IHC analysis in clinical samples. Among 251 cases of CRCs examined in this study, 157 cases (62.5%) displayed strong Cyr61 expression. The prevalence of Cyr61 expression in our study is lower than that reported by Landwa et al. [35]. This discrepancy could be attributed to either sample size or different primary antibodies used in the IHC analysis. Moreover, Cyr61 expression played some role in the progression of CRC in this study, as Cyr61 expression levels were higher in more advanced pT, pN, and TNM stages than in less advanced stages. Strong Cyr61 expression was associated with poor prognosis (p = 0.029), as determined by Cox hazard regression analysis. The survival analysis, performed using the Kaplan-Meier method, demonstrated that survival was significantly lower in patients with Cyr61 high expression than in patients with Cyr61 low expression (log rank test, p = 0.002). This study reports the first survival analysis involving the IHC evaluation of Cyr61 expression in CRC. The results of this study support the hypothesis that Cyr61 promotes cancer cell proliferation, invasion, and metastasis [4,5,26,27]. However, several lines of evidence support the hypothesis that Cyr61 can suppress tumour growth functioning as an antioncogene. Cyr61 inhibits NSCLC cell proliferation by upregulating p53, p21, and p130/Rb gene expression [15], suggesting that Cyr61 may induce senescence through a p53-/pRb-dependent pathway [36]. Likewise, overexpression of Cyr61 inhibits the proliferation of hepatocellular carcinoma cells, in part through p53 action [37]. Furthermore, overexpression of Cyr61 in endometrial carcinoma cells has been reported to decrease cell growth and increase apoptosis [16]. Additionally, Cyr61 expression in melanoma cells reduces tumour growth and metastasis and concomitantly increases apoptosis in tumours [38]. Thus, the role of Cyr61 in tumorigenesis may be dependent on cell type and context. In conclusion, the results of this study suggest that Cyr61 expression plays an important role in the carcinogenesis and progression of CRC, as well as in predicting patient survival. Further investigations may validate the potential utility of Cyr61 as a biomarker or therapeutic target against CRC.

## Conclusions

Our data suggest that Cyr61 expression in colorectal cancers is associated with patients survival and may thus serve as prognostic marker.

## Competing interests

The authors declare that they have no competing interests.

## Authors’ contributions

DJ, CJK, MSL, SL and MJB designed and supervised the study; DJ, CJK, MSL, SL, MJB, SP, HK, DP, HJK, YWJ, TSA, SSL and SHH acquired data and interpreted results; DJ, CJK, and MJB wrote the manuscript, but all authors helped with the drafting and editing. All authors read and approved the final manuscript.

## Pre-publication history

The pre-publication history for this paper can be accessed here:

http://www.biomedcentral.com/1471-2407/14/164/prepub
